# Unraveling Complex Interactions Mechanisms Linking PTSD and Chronic Diseases

**DOI:** 10.2174/011570159X392154250730072830

**Published:** 2025-08-21

**Authors:** Celina Cavalcante Muniz Gomes, Joelson Germano Crispim, Michelle Melgarejo da Rosa

**Affiliations:** 1 Center for Therapeutic Innovation, Suelly Galdino (NUPIT-SG), Recife, Brazil;; 2 Department of Biochemistry, Federal University of Pernambuco, Recife, Brazil

**Keywords:** Post-traumatic stress disorder, chronic diseases, treatment, stress-response, neurochemical factors, neurochemical pathways

## Abstract

Post-traumatic stress disorder (PTSD) is a chronic and multifactorial psychiatric condition that is often underdiagnosed, particularly when associated with chronic diseases (CDs). These conditions arise from complex interactions among psychosocial, socioeconomic, epigenetic, immune, metabolic, and neurobiological factors. Current treatment options for PTSD and CDs, whether isolated or comorbid, remain suboptimal. Addressing the bidirectional relationship between PTSD and CDs is a pressing global public health challenge, necessitating a deeper understanding of the underlying molecular mechanisms. This review examines the interplay of stress-response and neurochemical factors in PTSD and CDs, highlighting how maladaptive stress responses to trauma can disrupt neurochemical pathways, contributing to the development of CDs, and *vice versa*. Despite this, a significant gap exists in the number of *in vivo* model studies that adequately mimic the comorbid symptoms of PTSD and CDs, hindering progress in elucidating shared cellular and molecular pathways. This limitation restricts therapeutic advancements. Therefore, a comprehensive understanding of the neurobiological dysfunctions in the brain and their crosstalk with the immune, cardiovascular, and endocrine systems is critical. Such insights will pave the way for individualized treatment strategies tailored to the unique profiles of patients with PTSD associated with CDs.

## 1 INTRODUCTION

Post-traumatic stress disorder (PTSD) exemplifies how maladaptive fear responses can lead to persistent impairments in memory, learning, and cognition. It is characterized by intrusive thoughts, avoidance behaviors, and heightened arousal [1]. PTSD affects an estimated 3.9% of the global population at some point in their lives. This condition is associated with an increased risk of depression, suicidal ideation, and substance abuse [2]. Despite its high prevalence, PTSD often remains underdiagnosed, particularly in vulnerable populations [3]. Its complex clinical manifestations, which vary by symptom profile, present additional challenges to diagnosis and treatment. Current international guidelines recommend pharmacological treatments primarily designed for other psychiatric disorders, such as antidepressants, which rely on outdated evidence and fail to address critical symptoms like sleep disturbances [4]. The interplay between PTSD and Chronic Diseases (CDs) has garnered increasing attention due to their bidirectional relationship and shared pathophysiological pathways [5]. PTSD has been closely associated with oxidative stress [6] and inflammation [7], both of which are also implicated in CDs such as Parkinson’s disease (PD) [8], Alzheimer’s disease (AD) [9], diabetes [10], and cancer [11]. Furthermore, comorbid PTSD and CDs are known to disrupt the hypothalamic-pituitary-adrenal (HPA) axis, compromise the integrity of the blood-brain barrier (BBB), and exacerbate immune and inflammatory activation [12].

Despite these recognized overlaps, the neurobiological mechanisms underlying PTSD and its association with CDs remain poorly understood. This review provides an updated analysis of the stress-response and neurochemical factors linking PTSD and CDs. By examining shared mechanisms, it aims to illuminate novel therapeutic approaches that address the interconnected challenges of mental health and chronic illness.

## 2 METHODOLOGY

This study comprises a narrative review of the literature, based on a selection of clinical studies, behavioral studies, *in vitro* studies, and systematic reviews available in the PubMed, SciELO, and Scopus databases. The inclusion criteria included publications in English from the last 26 years that addressed limitations of currently used therapies and hypotheses of neurobiological mechanisms, immunoinflammatory processes, oxidative stress, and molecular markers related to PTSD, both in its isolated form and when associated with chronic diseases, such as autoimmune, cardiovascular, metabolic, and neurodegenerative diseases (NDs).

Data analysis considered experimental and clinical evidence, with emphasis on the identification of molecular biomarkers, diagnostic techniques, structural changes in the central nervous system, and clinical manifestations related to PTSD in different clinical contexts.

The main descriptors used in the search were: “PTSD” AND “neuroinflammation”, “oxidative stress”, “biomarkers”, “Alzheimer’s disease”, “HPA axis”, “Parkinson’s disease”, “Huntington’s disease”, “Chronic Pain”, “Diabetes”, “Cancer”, “alcohol use disorder”.

## 3 CLINICAL, NEUROBIOLOGICAL, AND IMMUNOMETABOLIC ALTERATIONS IN PTSD

Sigmund Freud initially defined trauma as an external violent attack that compromises the organism's integrity [13]. The development of PTSD involves multiple stages stemming from the interaction between external trauma and an individual's psychological response, underpinned by a hierarchical neurobiological framework. PTSD can result from diverse traumatic experiences, including childhood trauma, actual or threatened death, injury, sexual abuse, abuse of authority, natural or human-made disasters, war-torn displacement, domestic or community violence [3]. Underreporting is common, particularly in marginalized populations within middle- and low-income countries.

According to the Diagnostic and Statistical Manual of Mental Disorders, Fifth Edition (DSM-5), PTSD is categorized as a “Trauma- and Stressor-Related Disorder,” characterized by four core symptom clusters: intrusive memories and flashbacks of the traumatic event; avoidance of trauma-related cues and emotional numbing, negative mood and cognitive impairments, such as anhedonia and concentration difficulties; hyperarousal, including irritability, hypervigilance, exaggerated startle responses, and sleep disturbances, often involving nightmares.

**Fig. (1):**
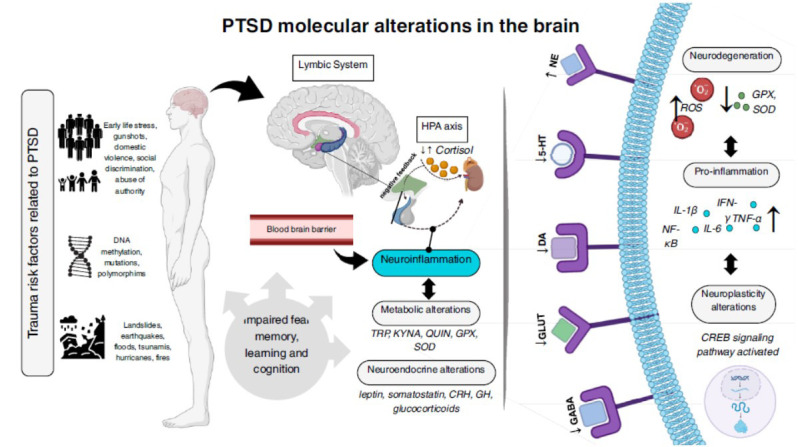
Molecular mechanisms behind Post-traumatic stress disorder (PTSD). PTSD’s development can be induced by human exposure to risk factors of physical, psychosocial, epigenetic, and biological nature, which lead to dysfunction in neurotransmission, metabolism, immune, and inflammatory responses to stress. The blood-brain barrier (BBB) can mediate the transport of high levels of pro-inflammatory cytokines to the central nervous system and may promote neurodegeneration. Some brain regions of the Limbic System (LS) and the hypothalamic-pituitary-adrenal (HPA) axis express increased levels of noradrenaline (NE) concentrations and decreased levels of gamma-aminobutyric acid (GABA), serotonin (5-HT), dopamine (DA), and glutamate (GLU). The agonism of noradrenergic, glutamatergic, GABAergic, and monoaminergic receptors induces biochemical cascades that activate cAMP response element-binding protein (CREB), the main transcription factor responsible for regulating gene expression in long-term memory fear conditioning. TRP: Tryptophan; GH: growth-hormone; TNF-α: Tumor Necrosis Factor-Alpha; IFN-γ: Interferon-Gamma; IL-1β: Interleukin-1-beta; IL-6: Interleukin-6; GPX: Glutathione Peroxidase; SOD: Superoxide Dismutase. Source: authors with BioRender.

Pro-inflammation in PTSD can affect the homeostasis of neuronal and glial populations and their associated glutamatergic and serotoninergic neurotransmission circuits in both central and peripheral brain regions (Fig. **1**). Key areas implicated in fear memory consolidation include the hippocampal CA1 region (contextual conditioning and inhibitory avoidance), the basolateral amygdala (inhibitory avoidance), and the prefrontal cortex, particularly the infralimbic region [1]. Patients with PTSD have demonstrated altered glutamatergic synaptic strength in the prefrontal cortex [14]. The influence of reduced glutamate neurotransmission on glial cells remains poorly understood in the literature. However, it is well established that pro-inflammation can alter glial cell function and glutamate regulation, leading to cell death through the activation of multiple avenues of excitotoxicity [15, 16].

Intensified pro-inflammatory responses can disrupt tryptophan (TRP) metabolism, a precursor to serotonin (5-HT). Altered TRP metabolism leads to increased levels of multiple glutamate-like metabolites, such as kynurenine metabolites, including kynurenic acid (KYNA) and quinolinic acid (QUIN), which modulate NMDA receptor activity and contribute to neurotoxicity [7]. Concurrently, reduced expression of antioxidant enzymes, including glutathione peroxidase (GPX) and superoxide dismutase (SOD), exacerbates mitochondrial production of reactive oxygen species (ROS), further promoting neurodegeneration [6, 17]. Glial cells could be potential therapeutic targets to enhance the release of glutamate and serotonin in the brain with PTSD.

Synaptic plasticity mechanisms underpin stress response mechanisms and involve both short-term and long-term potentiation (LTP). Dysfunction of synaptic plasticity is present in PTSD and can be influenced by inflammation [18]. A study showed that pro- and anti-inflammation significantly decreased LTP, brain-derived neurotrophic factor (BDNF) expression levels, and the phosphorylation of the AMPA (α-amino-3-hydroxy-5-methyl-4-isoxazolepropionic acid) receptor subunit GluR1, when compared to the control group [19].

The body's stress response primarily involves two axes: the sympathetic-adrenomedullary (SAM) axis and the HPA axis. The SAM axis mediates immediate responses through the release of noradrenaline (NE), while the HPA axis drives longer-term responses, including the release of glucocorticoids [20]. Corticotropin-releasing hormone (CRH) from the hypothalamus activates CRH type 1 receptors in the anterior pituitary, stimulating adrenocorticotropic hormone (ACTH) secretion and subsequent cortisol release from the adrenal cortex [21]. Cortisol supports the stress response by enhancing glucose availability, regulating immune function, and maintaining electrolyte balance. Additionally, neuroendocrine molecules such as ghrelin, growth hormone (GH), somatostatin, oxytocin, vasopressin, and leptin play regulatory roles in stress but remain underexplored in the context of PTSD [22]. There are persistent level variations and abnormal circadian secretion rhythms of cortisol in PTSD, dementia [23], osteoporosis, hypertension, and diabetes mellitus [24]. In a clinical study, the cortisol levels were higher in trauma-susceptible patients with PTSD than in trauma-resilient individuals with PTSD that fully recovered after similar trauma [25].

The peripheral immune response induced by trauma differs from infectious mechanisms, primarily due to a dysregulation of the HPA axis. This dysregulation contributes to a sustained pro-inflammatory state that fuels neuroinflammation, neurodegeneration, and altered synaptic plasticity. A key feature of this process is impaired glucocorticoid receptor (GR) sensitivity in limbic regions, which leads to cortisol resistance, hypercortisolism, and a failed negative feedback loop that promotes inflammation under chronic stress [26-29].

Crucially, the BBB plays a central role in mediating this neuroimmune interface. The BBB is a highly selective, three-dimensional structure composed of microvascular endothelial cells sealed by tight junctions, supported by astrocytic endfeet, pericytes, microglia, and the extracellular matrix [30]. Its integrity is essential for regulating molecular and cellular exchanges between the blood and the brain. However, peripheral inflammation and stress-induced cytokine release can disrupt BBB integrity, leading to increased permeability and facilitating neuroinflammatory cascades.

In PTSD, elevated levels of pro-inflammatory cytokines, such as TNF-α, IFN-γ, IL-1β, and IL-6, as well as CRP, have been consistently reported [7, 20, 31, 32]. These cytokines can cross the BBB through saturable transport systems or act on endothelial receptors to initiate signaling cascades that compromise tight junctions [33]. Notably, cytokines may enhance BBB permeability without even crossing into the brain, by disrupting endothelial tight junctions and altering transport pathways [34].

The consequence of such BBB breakdown is significant: peripheral immune cell infiltration becomes possible, including entry of leukocytes such as T cells, monocytes, and dendritic cells *via* the choroid plexus and cerebrospinal fluid (CSF) circulation [35]. These cells can access the brain parenchyma through diapedesis, a multistep process involving endothelial adhesion molecules and immune cell receptors [36], which further amplifies Central Nervous System (CNS) inflammation and may contribute to behavioral and cognitive deficits. Beyond cytokines, chemokines also mediate the attraction of immune cells to the CNS. Meta-analyses reveal altered levels of chemokines such as CCL2, CCL3, CCL4, CCL5, and CXCL12 in PTSD patients [37]. Notably, comorbid conditions like rheumatoid arthritis (RA) show elevated chemokines and cytokines when PTSD is present, suggesting an exacerbation of systemic inflammation in psychiatric comorbidity. This neuroinflammation is further driven by activated astrocytes and microglia, which respond to peripheral cytokines and altered neurotransmission by releasing additional inflammatory mediators. Mechanisms by which cytokines amplify their presence in the brain include direct transport, stimulation of circumventricular organs, afferent nerve signaling, and self-induced CNS release [36]. For instance, TNF-α can induce glial activation and neuronal apoptosis in the substantia nigra, raising the possibility that PTSD may exacerbate neurodegenerative conditions such as Parkinson’s disease [38].

Altogether, the disruption of the BBB serves not only as a marker of disease progression in PTSD and chronic diseases but also as a potential therapeutic target. Strategies aimed at preserving or restoring BBB integrity, such as pharmacological modulation of cytokine signaling, tight junction stabilization, or regulation of glial cells, may represent a promising avenue for mitigating neuroinflammation and its downstream effects on cognition and behavior in comorbid PTSD-CD states.

Stress responses vary among individuals, underscoring the importance of understanding how environmental factors interact with genes and their variants, particularly in cases of HPA axis hyporeactivity and dysregulation of fear memory consolidation [39]. Robust preclinical and clinical research has highlighted the role of immunophilins, especially FK506-binding proteins (FKBPs), in modulating central stress responses [40]. FKBP51, which is highly expressed in the hippocampus (HPC), functions as a co-chaperone that binds to glucocorticoid receptors (GRs), reducing their affinity for cortisol [40].

Elevated levels of FKBP51-GR protein complexes have been observed in fear-conditioned mice and the blood of PTSD patients, suggesting impaired GR nuclear translocation and reduced GR-mediated gene transcription [41]. An *in vivo* study suggested that disrupting the FKBP51-glucocorticoid receptor (GR) complex may alleviate acute aversive responses associated with fear conditioning [42]. Reduced GABAergic inhibitory function plays a key role in stress-induced fear memory expression and the generation of neuronal LTP, both fundamental to the neurobiological development of PTSD [43, 44]. FKBP51 expression also appears to be involved in an inflammation-induced stress-resilience phenotype. In this model, peripheral LPS stimulation activated GR signaling, which upregulated FKBP51, promoted neuroinflammation, suppressed c-Fos-induced neuronal activity, and increased GAD65 expression, enhancing GABA synthesis in the ventral CA1 subregion of the hippocampus [45]. In male mice, FKBP5 deficiency (which encodes FKBP51) attenuated LPS-induced TNF-α and IL-1β gene expression while enhancing the expression of the anti-inflammatory gene Arg-1 [45]. Another *in vivo* study showed that FKBP51 inhibition promotes stress resilience by preventing stress-induced social withdrawal and other stress-related behaviors in a chronic stress animal model [46]. Inhibition of FKBP51 by SAFit2 also promoted *in vitro* proliferation and neuronal differentiation of hippocampal neural progenitor cells, as well as neurite outgrowth [46]. Clinical studies have demonstrated that single-nucleotide polymorphisms (SNPs) in the FKBP5 gene increase susceptibility to PTSD [47-49]. SNPs such as rs9470080 and rs1360780 have been associated with impaired fear extinction, particularly in the amygdala [50]. In participants carrying FKBP5 SNPs rs1360780, rs3800373, and rs9296158, saliva samples showed associations with impaired immediate recall, lack of stress-enhanced long-term recall and recognition memory, and altered salivary cortisol levels—effects not observed in individuals without these polymorphisms [51].

SNP rs3800373 has been reported in African, American, and Caucasian populations, while rs9470080 has been identified in Caucasian PTSD groups, both associated with GR activation [52]. Moreover, SNPs rs3800373, rs9380526, rs9394314, rs2817032, and rs2817040 have been linked to increased severity of musculoskeletal pain following motor vehicle collisions and sexual assaults, traumatic events closely associated with PTSD [53-55].

In a neuroimaging study involving Han Chinese adults with PTSD following the loss of a child, the FKBP5 SNPs rs3800373, rs9296158, rs1360780, and rs9470080 were associated with decreased spectral power in the anterior cingulate cortex and increased power in motor/sensory areas, suggesting disturbances in emotional regulation pathways and heightened vigilance or sensitization to threat stimuli [56]. The relationship between FKBP polymorphisms, inflammation, and PTSD remains unexplored and warrants further investigation.

## 4 CHALLENGES AND THERAPEUTIC PHARMACOLOGICAL ALTERNATIVES FOR ADULTS WITH PTSD

Therapeutic approaches for PTSD are multifaceted, guided by evidence-based recommendations that prioritize symptom specificity and patient-centered care. First-line treatments often combine pharmacological interventions with trauma-focused psychotherapies, such as Cognitive Behavioral Therapy (CBT), Eye Movement Desensitization and Reprocessing (EMDR), Cognitive Processing Therapy (CPT), Prolonged Exposure Therapy (PET), and Imagery Rehearsal Therapy (IRT) [4]. While these psychotherapeutic approaches have shown significant benefits, pharmacological options for PTSD remain limited, often addressing only a subset of symptoms effectively.

Selective Serotonin Reuptake Inhibitors (SSRIs), including paroxetine, fluoxetine, and sertraline, are commonly prescribed. Though moderately effective for alleviating depressive and anxiety-related symptoms, SSRIs frequently fail to address core PTSD symptoms such as nightmares and hyperarousal [4]. Additionally, their side effects, including sexual dysfunction, sleep disturbances, and weight changes, can persist long after treatment cessation, posing challenges for long-term adherence [4].

In the last 50 years, there has been a sub-treatment and poor prognosis of humor disorders, parallel with the reduced financing initiatives for mental illness in comparison with other life-threatening diseases [57]. Psychedelic drug therapy presents promising antidepressant and anxiolytic effects, but also raises important safety concerns, including potential for abuse and long-term effects [58]. Such adverse outcomes are particularly associated with unsupervised use, inadequate preparation, or insufficient monitoring during clinical research [59]. The long-term psychological effects of psychedelics are theorized to stem from alterations in neural entropy, mystical experiences, emotional breakthroughs, and sustained changes in connectedness [58]. Nevertheless, an increasing number of randomized placebo-controlled trials demonstrate significant therapeutic effects in treatment-resistant depression [60, 61], social anxiety disorder [62], and preliminary outcomes for PTSD [63].

Randomized controlled trials have demonstrated the efficacy of MDMA-assisted psychotherapy in reducing PTSD symptom severity, improving psychological resilience, and alleviating functional impairments, particularly in severe cases [12, 64]. Studies by the Multidisciplinary Association for Psychedelic Studies (MAPS) have further highlighted the safety and superiority of MDMA-assisted psychotherapy over traditional pharmacological options like sertraline and paroxetine [65].

Cannabinoid-based treatments are also gaining attention for their role in PTSD management. Cannabidiol (CBD) oil has demonstrated anxiolytic effects, particularly in reducing fear responses and improving emotional regulation during traumatic memory recall [66]. The expression of cannabinoid receptors in key brain regions such as the medial prefrontal cortex (mPFC), hippocampus (HPC), and amygdala underscores their potential role in modulating learned fear and stress responses [67].

Despite the growing repertoire of therapeutic options, current treatments often fall short of addressing the complex and multifactorial nature of PTSD. The frequent comorbidity of PTSD with CDs further complicates treatment strategies. Shared molecular mechanisms, particularly those involving inflammatory regulation and neurotransmission, may underlie this overlap, emphasizing the need for integrated approaches. Understanding these connections is pivotal for improving diagnostic accuracy, monitoring, and the development of novel treatment modalities that target both PTSD and its associated comorbidities.

## 5 CHRONIC DISEASES AND PTSD

The decline of infectious diseases and the prevalence of CDs have defined the epidemiological landscape of the 20th century, influenced by consecutive industrial revolutions in Europe and global population aging [68, 69]. CDs often manifest silently in their early stages, presenting few or no symptoms, which complicates early diagnosis. Their lifelong nature demands costly medical technologies and extensive long-term care [68]. Survival and mortality rates in CDs are influenced by risk factors such as smoking, physical inactivity, and early exposure to harmful chemicals [69]. PTSD frequently co-occurs with CDs, either increasing their development risk or exacerbating existing symptoms. Shared inflammatory pathways and neurobiological alterations suggest a common pathophysiological framework between PTSD and CDs, offering intriguing opportunities for research. Understanding these intertwined mechanisms could pave the way for improved prognostic outcomes, innovative therapeutic strategies, and a better quality of life for patients enduring these debilitating conditions. Identifying overlapping neurobiological and inflammatory pathways in PTSD and CDs could also inform the development of integrated treatment approaches.

## 6 PTSD AND NEURODEGENERATIVE PROCESSES

NDs such as AD, PD, and HD are characterized by progressive cognitive and motor dysfunction, with aging and chronic inflammation as key risk factors (Table **1**). PTSD shares overlapping molecular mechanisms with NDs, including oxidative stress, apoptosis, neuroinflammation, disrupted circadian rhythms, and sleep disturbances [6, 17, 70]. These shared features suggest that PTSD may contribute to accelerated neurodegeneration and cognitive decline in aging individuals [64, 71].

For example, AD and PTSD lead to persistent activation of pro-inflammatory cytokines (*e.g*., IL-1β, TNF-α), and oxidative stress, which accelerate synaptic dysfunction and neuronal loss [72, 73]. Both conditions exhibit dysregulation of microglial function, with excessive activation leading to chronic inflammation and impaired clearance of amyloid-beta (Aβ) plaques in AD [72, 73]. Additionally, PTSD has been linked to an increased risk of late-life cognitive decline and may act as a predisposing factor for AD [9]. Tau hyperphosphorylation can be caused by neuroinflammation [74] and hippocampal atrophy [75], both of which are induced by HPA axis dysregulation [76]. It remains unclear which full signaling pathways interconnect these events, and these need to be further investigated.

Given these shared mechanisms, targeting neuroinflammatory pathways, such as modulating microglial activity or cytokine signaling, may offer novel therapeutic strategies to mitigate cognitive decline in both PTSD and chronic diseases.

Among genetic modulators, FKBP5 has emerged as a critical player in both stress-related psychiatric conditions and neurodegeneration. FKBP5 polymorphisms (*e.g*., rs1360780, rs9470080) have been linked to altered HPA axis function, poor stress resilience, and disrupted sleep patterns in PTSD [77], which in turn contribute to tau accumulation and increased dementia risk [14]. Preclinical studies also show FKBP51 interacts with Hsp90 to regulate tau stability and phosphorylation, and its knockdown reduces tau accumulation [78]. FKBP5 deletion in stress-exposed mice reduces corticosterone levels and promotes a pro-resilience phenotype [79].

Beyond tau, FKBP5 modulates autophagy and inflammatory responses relevant to NDs. In HD and PD models, FKBP5 inhibition enhances autophagy, reduces protein aggregation, and limits neuroinflammation [45, 80]. Additionally, FKBP5 interacts with the AKT-PHLPP2 signaling pathway, a pathway implicated in neuroprotection and protein homeostasis [81]. Impaired GR signaling due to FKBP5 dysregulation may also exacerbate neuroinflammation and dopaminergic loss in PD models [82, 83].

Although our review is narrative in style, we prioritized mechanistic studies with strong translational relevance when selecting literature on FKBP5 and psychedelics. Psychedelics were included based on emerging evidence supporting their neuroprotective effects, such as enhanced neurogenesis, anti-inflammatory activity, and modulation of the sigma-1 receptor [84, 85], which may indirectly counteract neurodegenerative processes associated with PTSD. Compounds such as ayahuasca and N, N-dimethyltryptamine (DMT) have been shown to suppress aversive memories *via* infralimbic cortex modulation and enhance neurogenesis in the dentate gyrus, potentially mediated by sigma-1 receptor (S1R) activation, a pathway implicated in PTSD [85], PD, and AD [86]. Similarly, β-carboline alkaloids from *Banisteriopsis caapi*, including harmine and tetrahydroharmine, inhibit monoamine oxidase A and B, reduce neuroinflammation, and enhance dopaminergic signaling in the striatum, a region crucial for motor function in PD [87-89]. Additionally, B. caapi-derived epicatechin and procyanidin exert antioxidant effects [88], further supporting their neuroprotective role. These findings underscore the need for further exploration of psychedelic-based therapeutics as a means to counteract neurodegenerative and PTSD-related cognitive impairments, ultimately offering a novel avenue for intervention in these disorders.

PTSD symptoms, such as memory impairment and sleep disturbances, often worsen with aging, contributing to accelerated cognitive decline [9]. Sleep disruptions and circadian rhythm alterations in PTSD patients are linked to the accumulation of tau and amyloid proteins, increasing the risk of tauopathies [90]. In humans, SNPs such as rs1360780, rs3800373, rs9296158, and rs9470080 in the FKBP5 gene have been associated with sleep disturbances, including respiratory events during REM sleep in patients with obstructive sleep apnea (OSA). They are considered risk factors for PTSD-related sleep issues [77]. These genetic variants have also been linked to suicidal behavior [91], emphasizing the broader impact of FKBP5 in psychiatric and neurodegenerative conditions. *In vitro* studies have demonstrated that FKBP51 stabilizes tau by interacting with Hsp90, thereby altering tau phosphorylation patterns and promoting microtubule stabilization [78]. FKBP51 knockdown in these studies reduced tau levels. Similarly, FKBP51 deletion in sleep-deprived, stress-exposed mice resulted in lower corticosterone levels, reduced HPA reactivity, and a pro-resilience sleep phenotype [79, 92].

Given these interconnected pathways, targeting FKBP5 signaling, sleep regulation, and neuroinflammation may offer new therapeutic strategies to prevent or delay neurodegeneration in individuals with PTSD. However, while preclinical evidence is promising, the therapeutic potential of psychedelics and tropomyosin receptor kinase B (TrkB) inhibitors remains preliminary and requires further validation in well-controlled clinical studies.

## 7 PTSD AND DIABETES

Diabetes involves altered glucose metabolism, oxidative damage to pancreatic β-cells, endothelial dysfunction, and dysregulation of the HPA axis, all of which contribute to the generation of ROS, influence intracellular signaling, and regulate apoptosis and immune responses [100]. Pathophysiological changes in diabetes include enhanced glucose uptake in adipose tissue, increased skeletal muscle mitochondrial function, and elevated serum expression of fibroblast growth factor 21 (FGF21) [96].

PTSD induces chronic dysregulation of the HPA axis, leading to sustained peripheral inflammation through the upregulation of pro-inflammatory cytokines such as TNF-α and IL-6 [101]. These inflammatory mediators activate intracellular pathways, including the c-Jun N-terminal kinase (JNK) and IκB kinase-β (IKK-β)/nuclear factor-κB (NF-κB) signaling cascades, contributing to the development of insulin resistance. Insulin resistance, a hallmark of type 2 diabetes mellitus (T2DM), disrupts glucose uptake, promotes oxidative stress, and alters hepatic lipid metabolism, culminating in hepatic steatosis and mitochondrial dysfunction [96, 100].

In experimental models, animals with preexisting diabetes exhibit exacerbated PTSD-like symptoms, including impaired memory, heightened anxiety-like behavior, and dysregulated stress responses [10]. Additionally, activation of mineralocorticoid receptors (MRs) in diabetic rats has been linked to the abnormal consolidation of aversive memories, further implicating HPA axis dysfunction in cognitive and emotional disturbances [1, 95].

Beyond glucose dysregulation, the chronic inflammation seen in PTSD may aggravate cardiovascular complications, as shown by myocardial lesions such as necrosis and fibrosis in rodent models exposed to chronic stress [102]. Emerging evidence also suggests that therapeutic interventions targeting the HPA axis and oxidative stress, such as cannabidiol (CBD) and pregabalin, may ameliorate both metabolic and neuropsychiatric symptoms by improving insulin sensitivity, reducing lipid peroxidation, and enhancing antioxidant defenses in the brain [103, 104].

Thus, PTSD-induced HPA axis dysregulation promotes a systemic pro-inflammatory state that impairs insulin signaling, perturbs hepatic and mitochondrial metabolism, and exacerbates the risk for metabolic disorders such as diabetes and cardiovascular disease.

## 8 PTSD AND CANCER

PTSD symptoms are frequently observed in cancer patients, particularly those with breast cancer [11] and hepatocellular carcinoma [105]. Nervous system alterations contribute to cancer progression *via* neuroplasticity and stress-induced activation of inflammatory pathways [86]. In individuals with PTSD and cancer, central and peripheral responses to stress exacerbate fear and anxiety, perpetuating PTSD symptoms and worsening patient outcomes [11].

PTSD may influence tumor progression through the brain-derived neurotrophic factor (BDNF) and TrkB pathways, which regulate synaptic plasticity, memory, and learning [1] (Table **1**). Upregulation of TrkB signaling has been implicated in tumor growth in breast cancer and gliomas [98], as well as hepatocellular carcinoma [98]. In gliomas, BDNF-TrkB-MAPK signaling regulates malignant synaptic changes, linking PTSD-related neuroplasticity to tumor dynamics [98]. Altered BDNF-TrkB signaling in the hippocampus, amygdala, anterior cingulate cortex, ventromedial prefrontal cortex, and nucleus accumbens may contribute to PTSD predisposition and maintenance [106, 107]. These studies are still in the early stages, but there is growing preclinical evidence showing the potential therapeutic role of TrkB inhibitors.

## 9 PTSD AND ALCOHOL ABUSE DISORDERS

Concurrent treatments targeting both PTSD and other diseases have shown promising results in mitigating symptoms. For instance, alcohol use disorder (AUD) can interfere with PTSD treatment by promoting disengagement from traumatic memory processing and slowing therapeutic efficacy [108]. However, combined approaches, such as CPT alongside topiramate pharmacotherapy, have demonstrated improved outcomes. In one case, this combination reduced alcohol dependence and lessened the intensity of reactions to traumatic memories in a patient with PTSD and AUD [108].

Topiramate, an anticonvulsant that inhibits GABA and AMPA/kainate glutamatergic receptors, enhances fear memory consolidation, potentially enabling patients to process traumatic memories and develop tolerance to emotional distress [108]. A clinical trial also found that topiramate reduced hyperarousal symptoms and alcohol consumption in PTSD patients with AUD [109].

## CONCLUSION AND FUTURE PERSPECTIVES

Despite recent advancements, significant challenges persist in understanding and addressing the complex interplay between PTSD and chronic diseases (CDs) such as cancer, neurodegenerative disorders (NDs), and metabolic conditions. Currently, no pharmacological therapies effectively mitigate the progression of CDs or manage PTSD symptoms. Comorbid PTSD accelerates neurodegeneration and cognitive decline, particularly in individuals with preexisting CDs, emphasizing the urgent need for innovative treatments. Preclinical studies have identified potential therapeutic compounds, such as DMT, harmaline, harmine, tetrahydroharmine, epicatechin, and procyanidin (derived from Ayahuasca), which demonstrate neuroprotective effects and could facilitate fear memory extinction. However, their efficacy in complex disease models remains underexplored. The absence of comprehensive *in vivo* models that replicate the overlapping symptoms of PTSD and CDs, particularly in cancer and neurodegeneration, hinders translational progress. These models need to incorporate behavioral, neuroinflammatory, and molecular dimensions to reflect the dynamic interactions between these conditions better. Furthermore, clinical trials often suffer from limited sample sizes and a lack of long-term follow-up, which weakens the evidence base and limits the identification of personalized treatment strategies. The insufficient exploration of how PTSD interacts with other systems—such as immune, cardiovascular, and endocrine—compounds these challenges. A deeper understanding of these cross-systemic interactions is vital for addressing the multifaceted impacts of PTSD in patients with CDs. To advance the field, future studies should prioritize developing more sophisticated animal and *in vitro* models that integrate PTSD-like symptoms with specific CD pathologies, such as glioma progression or metabolic dysfunction. Achieving this requires cross-disciplinary collaboration between neuroscientists, immunologists, and oncologists to create models that reflect the interconnected pathophysiology of these conditions. Additionally, leveraging insights into the neurobiological dysfunctions of PTSD and CDs will be key in designing individualized therapeutic interventions. These approaches may involve combining pharmacological agents (*e.g*., TrkB modulators) with psychotherapeutic methods and non-invasive neuromodulation techniques, such as transcranial magnetic stimulation (TMS). Clinical research should also diversify participant populations to include those affected by trauma from non-military contexts, as well as individuals with pre-existing CDs. Longitudinal studies with adequate sample sizes are crucial for evaluating the efficacy and safety of emerging therapies and ensuring their broad applicability. A comprehensive understanding of the relationship between PTSD and CDs demands a systems biology approach, integrating molecular, behavioral, and clinical insights. The use of advanced computational models and AI-driven analytics could expedite the identification of biomarkers and predict treatment responses. Expanding international collaborations and funding will also play a crucial role in filling current knowledge gaps and fostering innovative therapies. By addressing these challenges, the field can make significant strides toward providing personalized, comprehensive care, ultimately improving the prognosis and quality of life for individuals suffering from PTSD and CDs.

## Figures and Tables

**Table 1:** Summary of common genes or proteins related to PTSD and CDs.

**Proteins Altered**	**Disease Symptoms Related**	**Function Related**	**References**
FKBPs	PTSD, HD, CP, tauopathies, diabetes, and obesity	The polymorphisms rs1360780, rs3800373, rs9296158, and rs9470080 within the FKBP51/FKBP5 gene can increase the risk of PTSD’s development.	[48]
		Reduced levels of FKBP5 in HD R6/2 and zQ175 mouse models and human HD isogenic neural stem cells and medium spiny neurons derived from induced pluripotent stem cells.	[80]
		FKBP51-null mice exhibited resistance to weight gain and hepatic steatosis, along with reduced white adipose tissue and increased brown adipose tissue.	[93]
		FKBP52 may enhance tau-mediated neuronal dysfunction by activating a caspase-dependent pathway, thereby contributing to memory and learning impairments in transgenic mice.	[94]
FGF21	PTSD and diabetes	Mineralocorticoid receptor activation in diabetic rats disrupts the overconsolidation of aversive memory.	[95]
		In mice exposed to chronic stress, characterized by increased plasma corticosterone and hepatic insulin resistance, plasma FGF21 levels were significantly elevated and correlated with the expression of genes involved in fatty acid oxidation and the formation of brown-like adipocytes.	[96]
TrkB signaling pathway	PTSD, AD, glioma, and breast cancer	TrkB receptors modulate specific phases of fear learning and amygdalar synaptic plasticity through two main phosphorylation docking sites: 1) Y816F point mutation impairs acquisition of FC, amygdalar synaptic plasticity, and CaMKII signaling at synapses; 2) Y515F point mutation affects consolidation but not acquisition of FC to tone, and also alters AKT signaling.	[97]
		TrkB signaling to CAMKII, *via* BDNF, promotes AMPA receptor trafficking to the membranes of glioma cells, enhancing glutamate-evoked currents in malignant glioma cells and supporting tumor proliferation through synaptic plasticity mechanisms.	[98]
		TrkB promotes the tumorigenicity and metastasis of breast cancer cells by suppressing Runx3 or Keap1.	[99]

**Abbreviations**: PTSD: Post-traumatic Stress Disorder; HD: Huntington Disease; AD: Alzheimer’s Disease; DP: Parkinson’s Disease; CP: Chronic Pain; FGF21: Fibroblast growth factor 21; BDNF: Brain Derived Neural Factor; TrkB: Tropomyosin receptor kinase B; CAMKII: Ca^2+^/calmodulin-dependent protein kinase; FC: Fear Conditioning. Source: authors.

## LIST OF ABBREVIATIONS

5-HT: Serotonin
AD: Alzheimer’s Disease
AMPA: α-amino-3-hydroxy-5-methyl-4-isoxazolepro-pionic Acid
AUD: Alcohol Use Disorder
Aβ: Amyloid-beta (Aβ)
BBB: Blood-brain Barrier
BDNF: Brain Derived Neural Factor
CAMKII: Ca^2+^/Calmodulin-dependent Protein Kinase
CBD: Cannabidiol
CBT: Cognitive Behavioral Therapy
CDs: Chronic Diseases
CNS: Central Nervous System
CPT: Cognitive Processing Therapy
CREB: Element-binding Protein
CRH: Corticotropin-releasing Hormone
CSF: Cerebrospinal Fluid
DA: Dopamine
DMT: N,N-dimethyltryptamine
DSM-5: Statistical Manual of Mental Disorders, Fifth Edition
EMDR: Eye Movement Desensitization and Reprocessing
FC: Fear Conditioning
FGF21: Fibroblast Growth Factor 21
FKBPs: FK506-binding Proteins
GABA: Gamma-aminobutyric Acid
GH: Growth Hormone
GLU: Glutamate
GPX: Glutathione Peroxidase
GR: Glucocorticoid Receptor
HPA: Hypothalamic-pituitary-adrenal
HPC: Hippocampus
IFN-γ: Interferon-gamma
IKK-β: IκB Kinase-β
IL-1-β: Interleukin-1-beta
IL-6: Interleukin-6
IRT: Imagery Rehearsal Therapy
JNK: c-Jun N-terminal Kinase
KYNA: Kynurenic Acid
LS: Limbic System
LTP: Long-term Potentiation
MAPS: Multidisciplinary Association for Psychedelic Studies
mPFC: medial Prefrontal Cortex
MRs: Mineralocorticoid Receptors
NDs: Neurodegenerative Diseases
NE: Noradrenaline
NF-κB: Nuclear Factor-κB
PD: Parkinson’s Disease
PET: Prolonged Exposure Therapy
PTSD: Post-traumatic Stress Disorder
QUIN: Quinolinic Acid
ROS: Reactive Oxygen Species
SAM: Sympathetic-adrenomedullary
SOD: Superoxide Dismutase
SSRIs: Selective Serotonin Reuptake Inhibitors
T2DM: Type 2 Diabetes Mellitus
TMS: Transcranial Magnetic Stimulation
TNF-α: Tumor Necrosis Factor-alpha
TrkB: Tropomyosin Receptor Kinase B
TRP: Tryptophan
